# Population Characteristics and the Nature of Egg Shells of two Phthirapteran Species Parasitizing Indian Cattle Egrets

**DOI:** 10.1673/031.010.14123

**Published:** 2010-09-27

**Authors:** Aftab Ahmad, Vikram Khan, Smita Badola, Gaurav Arya, Nayanci Bansal, A. K. Saxena

**Affiliations:** Department of Zoology, Government Raza Postgraduate College, Rampur, Uttar Pradesh, 244901, India

**Keywords:** Amblycera, lschnocera, *Ardeicola expallidus*, *Ciconiphilus decimfasciatus*

## Abstract

The prevalence, intensities of infestation, range of infestation and population composition of two phthirapteran species, *Ardeicola expallidus* Blagoveshtchensky (Phthiraptera: Philopteridae) and *Ciconiphilus decimfasciatus* Boisduval and Lacordaire (Menoponidae) on seventy cattle egrets were recorded during August 2004 to March 2005, in India. The frequency distribution patterns of both the species were skewed but did not correspond to the negative binomial model. The oviposition sites, egg laying patterns and the nature of the eggs of the two species were markedly different.

## Introduction

Certain workers have indicated the population characteristics of Phthiraptera on selected avian hosts. Saxena et al. (2007) and Gupta et al. (2007) have noted the prevalence, intensity of infestation and the applicability of the negative binomial model in the frequency distribution patterns of twelve phthirapteran species occurring on house sparrows, Indian parakeets, common mynas, white breasted kingfishers and red avadavats, in the district of Rampur Uttar Pradesh, India. Rekasi et al. (1997) noted the frequency distribution of 15 species of avian lice and also reviewed 12 previously described distributions. Rozsa (1997) examined the ecological factors expected to determine the abundance of lice on birds. Reiezigel et al. (2005) recommended the determination of crowding indices to analyze parasite populations.

There are no reports on the population levels of Phthiraptera parasitizing cattle egrets. The present report furnishes information on the prevalence, intensities of infestation and the frequency distribution patterns of two phthirapterans infesting this bird. Furthermore, information on the egg laying sites, patterns of oviposition and egg morphology of both the species are also described.

## Materials and Methods

Seventy cattle egrets (*Bubulcus ibis* L.) were trapped live during August 2004 - March 2005, in the district of Rampur. After tying the legs, each bird was critically examined (with the help of magnifying lens). Louse free birds were immediately released and the infested birds subjected to delousing using “fair isle” method (Saxena et al. 2007). Lice were transferred to 70% alcohol and separated by species, stage and sex, for further analysis. The data were used for recording the prevalence, mean intensity, sample mean abundance and variance to mean ratio of the louse population. The exponent (k) of the negative binomial distribution and index of discrepancy (D) were estimated with software developed by Rozsa et al. (2000). The goodness of fit between observed and expected frequencies (negative binomial) were determined by the χ^2^ test. Birds heavily infested with each species were critically examined to record the number of eggs laid on the feathers of different regions of the body. Certain egged feathers were gently cut to record the patterns of oviposition, under stereozoom trinocular microscope. A few eggs were teased out and examined by SEM using the methods described by Gupta et al.. (2009)

## Results

One ischnoceran species, *Ardeicola expallidus* (Blagoveshtchensky), (Phthiraptera: Philopteridae) and one amblyceran species, *Ciconiphilus decimfasciatus* (Boisduval and Lacordaire) (Menoponidae) were recorded from seventy cattle egrets during survey work.

### Population characteristics

*A. expallidus:* A total of 633 specimens were collected from 12 infested birds (prevalence 17.2 %; sample mean abundance - 9.0; range of infestation, 14 – 120; mean intensity - 52.8). Frequency distribution pattern was skewed (variance to mean ratio - 71.5) and the observed frequencies failed to correspond to the negative binomial distribution (χ^2^ = 64.7, P > 0.05; exponent of negative binomial 0.04; D of poulin - 0.88). Females outnumbered the males in natural population (male, female ratio - 1:1.2) while nymphal population dominated over the adults (adult, nymph ratio - 1:1.2). The ratio of the nymphal population (first, second and third nymphal instars) remained 1:0.7:0.5.

*C. decimfasiatus*: Total numbers collected from 29 infested hosts was 2993 (prevalence 41.4 %; sample mean abundance - 42.8; range of infestation, 2 – 241 and mean intensity 103.2). Frequency distribution was hollow curve type (variance to mean ratio - 126.9). The negative binomial was not found to be a good fit (χ^2^ = 35.9, P > 0.05; exponent of negative binomial - 0.1; D of poulin - 0.76). The sex ratio was female biased (male, female ratio - 1:1.2). The nymphal population was slightly greater than the adult population (adult, nymph ratio - 1:1.1). The ratio of the three nymphal instars was 1: 0.8:0.5

### Egg lying site and pattern

The ischnoceran louse, *A. expallidus* showed restricted oviposition sites on the host body (73% wings, 12% tail, 9% abdomen, 3% breast, 2% nape and 1% neck). The eggs were laid inside the furrows, between the barbs and near the rachis (Plate I, 1). As many as seven eggs have been found lined one behind the other in a single furrow. The egg was inclined at 30–50°, with respect to the rachis. The maximum number of eggs encountered on a single feather was 180.

The amblyceran louse, *C. decimfasciatus* exhibited more or less widespread oviposition sites on the host body (45% breast, 31% abdomen, 9% back, 8% legs, 4% neck, 2% nape and 1% tail). Eggs were laid on the lateral plumulaceous portion of the vane. This louse showed a tendency to lay fresh eggs near the already laid eggs. Thus, eggs were laid in groups in somewhat “grape bunch” pattern (Plate I, 2, 3). Eggs were inclined at 25–40° and were glued at the rear end. More than 500 eggs were counted on a single feather.

### Egg morphology

The egg chorion of *A. expallidus* (length 0.9 – 1.0 mm, width 0.17 – 0.19 mm) bears very prominent elongated hexagonal ridges (Plate II, 1). Opercular disc of the egg also bears similar (but faint) ridges. A thick rod like small polar thread arises from the lateral side of the operculum (Plate II, 3, 4). The stigma has a rosette-like (0.021 mm in diameter) in appearance (Plate II, 5). The egg of *C. decimfasciatus* is ovoid in shape (0.6 – 0.7 mm in length and 0.18–0.20 mm in width) (Plate II, 2). The egg chorion is smooth (i.e., devoid of sculpturing /ornamentation). The operculum is hat-shaped and lacks polar thread (Plate II, 6). The opercular disc bears hexagonal marks. Eleven to fifteen button shaped micropyles are lined along the opercular rim. The stigma has a beehive-like appearance (0.033 mm in diameter).

## Discussion

Two phthirapteran species (*A. expallidus* and *C. decimfasciatus*) are known to occur on cattle egrets (Price et al. 2003). The mean intensity of *C. decimfasciatus* on Indian cattle egrets was very high (103.2). This amblyceran louse is haematophagous in nature and the crop of adults and nymphs were found full of host blood. The haematophagous lice can cause skin lesions that can be a probable site of secondary infection, irritation, restlessness, reduced egg production and weight loss in the infested hosts (Wall and Shearer 2001; Mullen and Durden 2002). Furthermore, they may also act as reservoir and transmitter of pathogens responsible for infectious diseases (Price and Graham 1997).

The prevalence of two lice on cattle egrets was not as high (17.2 – 41.4 %) in comparison to that of other species (12.5 – 97.5 %) (Saxena et al. 2007; Rekasi 1997; Rozsa et al. 1997). However, the mean intensity appeared to be high (52.8, *A. expallidus* and 103.2, *C. decimfasciatus*) in comparison to other species (0.26 – 37.7) examined by the above mentioned workers.

Out of the 27 cases analyzed by Rekasi et al. (1997) the frequency distribution of 19 species conformed to the negative binomial model. The negative binomial was found to be a good fit in only one case (out of 12 species) by Saxena et al. (2007) and Gupta et al. (2007). In the present case the frequency distribution of both the species was aggregated but failed to correspond to the negative binomial model. However, the significance of the frequency distribution lies not in the statistical patterns itself, but in the underlining ecological factors that are responsible for the generation of non-random distribution of parasite (Randolph 1975). Workers like Crofton (1971) and Randolph (1975) have postulated a series of situations which might give rise to the contagious distribution of parasites (i.e. non-random distribution of host, resistance to infestation by previously infested hosts, seasonal variation in infestation level of parasites and non-random differences in behavior and physiology such as breeding success and moult etc.) that are related to population. It is difficult to postulate the factor that might account for the observed distribution of parasites on cattle egrets. Since avian lice exhibit seasonal variation in population levels, the population of nymphs may vary from to time. Apart from seasons, many other factors can affect the population structure (Marshall 1981).

**Plate I:**
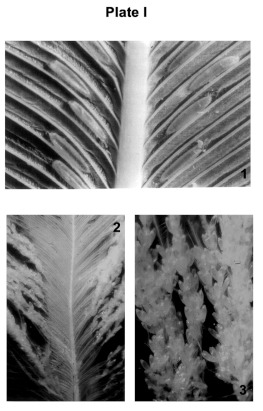
1. Feather bearing eggs of *Ardeicola expallidus* 2. Feather bearing eggs of *Ciconiphilus decimfasciatus* 3. Enlarged view of 2.

**Plate II:**
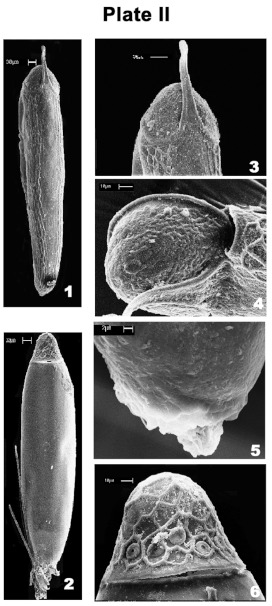
1. SEM of the eggshell of *Ardeicola expallidus* 2. SEM of the eggshell of *Ciconiphilus decimfasciatus* 3. Enlarged view of the opercular end of *Ardeicola expallidus* showing rod-like polar thread 4. Another enlarged view of the opercular disc of *Ardeicola expallidus* 5. Enlarged view of rear end of the eggshell of *Ardeicola expallidus* showing the nature of stigma 6. Enlarged view of the opercular end of *Ciconibhilus decimfasciatus.*

The nymph population had a slight edge over the adult population in the case of 9 species (out of 10) examined by Saxena et al. (2007) while adults dominated over nymphal population in the case of 2 species studied by Gupta et al. (2007). In the present case, the proportion of nymphs remained slightly higher than adults (sign of expanding population).

In the case of both the cattle egret lice, the sex ratios were female biased, as expected. In all of the 12 species examined by Saxena et al. (2007) and Gupta et al. (2007), the male, female ratio remained 1:1.1 to 1:1.65. Sampling bias (due to small size of males) and unequal longevity of the two sexes have been considered responsible for sex ratio biases (Marshall 1981). Furthermore, the avian lice exhibit considerable diversity with respect to the pattern of egg laying on body feathers (Marshall 1981). The same has been found to be true in the case of the two avian lice infesting cattle egrets.

The avian lice exhibit certain distinctive features (on or within chorionic shell) in the form of markings/ sculpturings/ ornamentation/ projections on the eggshell. Balter (1968 a and b) remarked that louse egg morphology could be used as a guide to louse taxonomy. Kumar et al. (2004) noted the eggshell markings of 3 species of *Lipeurus* differed (*L. heterographus* has distinct hexagonal ridges; *L. lawrensis tropicalis* has a chorion pitted with faint hexagonal ridges; *L. caponis* has granular protuberances). Gupta et al. (2007) showed that the egg shell of selected species of *Menacanthus* differed in number, location and nature of apophyses. Likewise, the eggshells of selected species of *Brueelia* differ in the presence of polar thread as well as the number and disposition of micropyles. However, the eggshells of other species of *Ardeicola* and *Ciconiphilus* have not yet been studied to provide the comparison.
